# Towards Robot-Assisted Retinal Vein Cannulation: A Motorized Force-Sensing Microneedle Integrated with a Handheld Micromanipulator †

**DOI:** 10.3390/s17102195

**Published:** 2017-09-23

**Authors:** Berk Gonenc, Jeremy Chae, Peter Gehlbach, Russell H. Taylor, Iulian Iordachita

**Affiliations:** 1 Computer Integrated Surgical Systems and Technology Engineering Research Center (CISST ERC), Johns Hopkins University, Baltimore, MD 21218, USA; rht@jhu.edu (R.H.T.); iordachita@jhu.edu (I.I.); 2 Wilmer Eye Institute, The Johns Hopkins School of Medicine, Baltimore, MD 21287, USA; jchae3@jhmi.edu (J.C.); pgelbach@jhmi.edu (P.G.)

**Keywords:** force sensing, fiber Bragg grating, retinal vein cannulation

## Abstract

Retinal vein cannulation is a technically demanding surgical procedure where therapeutic agents are injected into the retinal veins to treat occlusions. The clinical feasibility of this approach has been largely limited by the technical challenges associated with performing the procedure. Among the challenges to successful vein cannulation are identifying the moment of venous puncture, achieving cannulation of the micro-vessel, and maintaining cannulation throughout drug delivery. Recent advances in medical robotics and sensing of tool-tissue interaction forces have the potential to address each of these challenges as well as to prevent tissue trauma, minimize complications, diminish surgeon effort, and ultimately promote successful retinal vein cannulation. In this paper, we develop an assistive system combining a handheld micromanipulator, called “Micron”, with a force-sensing microneedle. Using this system, we examine two distinct methods of precisely detecting the instant of venous puncture. This is based on measured tool-tissue interaction forces and also the tracked position of the needle tip. In addition to the existing tremor canceling function of Micron, a new control method is implemented to actively compensate unintended movements of the operator, and to keep the cannulation device securely inside the vein following cannulation. To demonstrate the capabilities and performance of our uniquely upgraded system, we present a multi-user artificial phantom study with subjects from three different surgical skill levels. Results show that our puncture detection algorithm, when combined with the active positive holding feature enables sustained cannulation which is most evident in smaller veins. Notable is that the active holding function significantly attenuates tool motion in the vein, thereby reduces the trauma during cannulation.

## 1. Introduction

### 1.1. Motivation

Retinal vein occlusion (RVO) is one of the most common retinal vascular diseases affecting approximately 16.4 million people worldwide [1], with a prevalence of 1.8% and 0.5% for central and branch retinal veins, respectively [2]. RVO occurs when there is arterial stiffening/thickening of the crossing artery’s wall, low flow, hyper-coagulability, or thrombosis in the central retinal vein or its branches. The anatomic result of retinal vein occlusion is dilation, tortuosity and intraretinal hemorrhage. In many cases, the resulting ischemia leads to macular edema and decreased vision. Other serious complications include neovascularization, vitreous hemorrhage, and retinal detachment [3,4]. Patients with RVO are often experience blurred or distorted vision, and in severe cases permanent vision loss [5].

The available treatments for RVO include, but are not limited to, photocoagulation, hemo-dilution, radial optic neurotomy, vitrectomy and intravitreal injections. The predominant therapy at this time is serial intravitreous injections of anti-VEGF agents with the intent of limiting the damage induced by the occlusion, rather than resolving it [6,7,8,9,10]. Retinal vein cannulation (RVC) is a still investigational surgical procedure proposed to treat RVO by direct therapeutic agent delivery methods. The procedure involves three main steps: (1) accurately bringing a sharp tipped cannula onto the occluded retinal vein, (2) puncturing through the vein wall and precisely halting the cannula tip at the right depth, and (3) advancing and maintaining the cannula inside the vein for several minutes, during which a therapeutic agent e.g., tissue plasminogen activator (t-PA) [11] or ocriplasmin [12] or other is delivered to dissolve the thrombus. This is a very demanding and risky procedure because of the small size and fragility of retinal veins—especially if the occlusion is in a branch retinal vein (typically Ø < 200 μm) rather than the central vein [13].

### 1.2. Background

Several studies have explored cannulation of retinal vessels using a variety of in vivo models. In porcine eyes, it was shown that puncturing larger proximal vessels is technically easier than piercing the smaller veins more distal to the optic nerve after they have branched [14]. Nevertheless, when a small branch vein is occluded, cannulation of a nearby larger vessel instead is thought to diminish the effectiveness of the treatment [15]. To easily pierce the retinal vasculature and enable injection directly into such small branch veins, glass micropipettes with very fine sharp tips have been used [16,17,18]. However, the transparent small tips are hard to visualize under the microscope and the fragility of glass raises valid safety concerns. An alternative is to use stainless steel microneedles (Ø 50 µm) which have been used to inject balanced saline solution to dissolve a clot in prior work [19], and to mechanically fragment thrombi by advancing a soft thin (Ø 100 µm) wire through the lumen of a 37-gauge needle [20]. Tests on porcine eyes showed the feasibility, and this was further supported by successful clinical demonstrations in human retinal veins with few intraoperative and postoperative complications [21]. Using a curved cannula or an angulated tip accessing the vessel at a strategic angle has been found to be easier and safer. However, there were challenges in introducing the bent tip into the eye through the sclera, requiring either a modified incision or accommodating trocar through the sclera. In order to enhance the intraocular distal dexterity, a stent deployment unit with adjustable approach angle via concentric tubes was devised, and the optimal approach angle while cannulating vasculature on the chorioallantoic membrane (CAM) of fertilized chicken eggs—an accepted in vivo model for RVC studies [22]—was explored. This work revealed an optimum range of 25–35°, which is not easy to achieve via a straight instrument [23]. Flexible prebent stainless steel microneedles, which are normally held straight inside a protective shaft to be more easily introduced into the eye, have been developed. When deployed, these microneedles assume the optimum angle as they approach the retinal surface [24,25].

Despite the aforementioned progress effective and safe RVC remains a procedure at the limits of human performance. Three main challenges remain: first, a cannula thin enough to be inserted into the retinal vein (Ø < 100 µm) that can also withstand the forces of venipuncture and remains easily visible under the microscope is required. This challenge has partially been resolved by using stainless steel microneedles that replace previous transparent and fragile glass micropipettes. The second challenge is to control physiological hand tremor of the vitreoretinal surgeon, the normal scale of which is comparable in amplitude to the size of retinal veins [26]. Hand tremor limits precise targeting of retinal veins and also limits the ability to maintain the cannula within the lumen for the period of drug delivery [11]. In order to provide a more precise tool manipulation in vitreoretinal surgery, various robotic systems have been developed during the past 20 years [27]. These research platforms include teleoperated devices [28,29,30,31,32], cooperatively-controlled systems [33,34], injectable ocular microrobots [35,36], or handheld tools [37,38,39,40,41,42,43], and mostly shared the common goal of suppressing involuntary components of motion, such as the physiological hand tremor of the operator. Studies have now shown improved precision during procedures performed in artificial phantoms and animal models [18], and the potential to facilitate a safer operation on the extremely delicate retinal tissues during human surgery. However, it wasn’t until recently that any of these systems appeared in an actual retinal microsurgery. In September 2016, using the Preceyes Surgical System, a motion-scaling tremor-suppressing teleoperated robot [30], surgeons at Oxford’s John Radcliffe Hospital performed the world’s first robot-assisted vitreoretinal surgery for a membrane peeling operation [44]. In January 2017, eye surgeons at University Hospitals Leuven were the first to demonstrate robot-assisted RVC using another system to inject t-PA into the patient’s occluded retinal vein [45]. These two major proof of principal steps show the potential benefits and feasibility of robot-assisted tool manipulation during vitreoretinal surgery. Nevertheless, they do not address the third challenge in RVC, which stems from the fact that cannulating retinal veins occurs at forces that are almost imperceptible to humans. This fact makes the instant of venous puncture nearly imperceptible limiting the unassisted human effort to halt needle advancement and appropriately begin t-PA injection at the correct depth, leading to double punctures and undesired subretinal injections during in vivo trials [18]. Unlike cannulating retinal veins, conventional venipuncture occurs on larger structures and the resulting tactile forces are both perceptible and familiar to experienced phlebotomists. Specifically the clinician can feel the moment of vessel puncture [46]. Tests on the CAM model [29] have confirmed that most RVC forces lie below the human perception threshold, yet there is still a sharp force drop upon venous puncture, as observed in conventional venipuncture [47]. Continual monitoring of these very fine tool-tissue interactions via a very sensitive force sensor therefore has the potential to inform the operator of the moment of venous puncture during RVC and could also identify a second puncture indicating exit from the vessel.

Prior studies proposed several methods to quantify tool-tissue interaction forces in microsurgery and minimally invasive surgery (MIS). Semiconductor strain gauges were used to sense forces on robotic microgrippers [48]. The resulting geometry occupied an overall length of 17 mm, width of 7.5 mm and thickness of 0.4 mm. Micro-newton scale force sensing for safely manipulating cells was achieved by using diffractive optical MEMS encoders on a silicon-nitride probe [49]. To monitor tool-tissue interaction forces in MIS, a sensor was developed for use on laparoscopic instruments, with an outer diameter of 5 mm and based on intensity-modulated fiber optics to capture the three-dimensional forces with a resolution of 0.01 N [50]. Another fiber-optics based force sensor was shown to measure the tri-axial tool-tissue interaction forces with a resolution of 0.02 N during tissue palpation in MIS [51]. Using strain gauges mounted on a Steward platform, a 6-degree of freedom (DOF) force-sensing forceps was designed again for MIS applications, which provided a force resolution of 0.05 N for transverse loads and 0.25 N for axial loads [52]. In microsurgery, specifically in stapedectomy, integration of fiber Bragg grating (FBG) strain sensors on a micro-forceps enabled the first time quantification of forces involved in crimping of a stapes prosthesis, which ranged from 2.4 N to 5.2 N [53]. Based on a monolithic structure flexure and photo-sensors, a compact axial force sensor was developed providing a force resolution of 0.48 N [54], and was shown to properly work with a handheld robotic device to maintain a fixed contact force (200 and 400 mN) at the tool tip [42]. Recently, a strain-gauge-based force-sensing bipolar forceps was developed and used to measure axial and planar forces ranging up to 1.20 N during neurosurgical tasks in cadaveric brain experiments [55].

The force sensing methods in the above studies have several limitations that impede their application to vitreoretinal surgery. In order to measure the very fine tool-tissue interaction forces, force-sensing instruments with millinewton resolution and accuracy are required. This requirement was addressed by a miniature tri-axial force sensor based on strain gauges, which was designed to be mounted on the handle of a microsurgical instrument [56]. However, in retinal microsurgery, the tools are inserted through an incision in the sclera, and the contact forces at the insertion port are considerably larger than the tissue manipulation forces at the tool’s tip. Therefore, a handle mounted force sensor is not practical for vitreoretinal surgery, as the measurements would involve not only the tool-tissue interaction forces but also the adverse effect of frictional forces at the insertion port [57]. This necessitates positioning the force sensor proximal to the tool tip inside the eye, which imposes strict dimension constraints as well as biocompatibility, sensitivity and safety requirements, and hence limits the available sensors for this use.

To provide force feedback in retinal microsurgery, our team has developed force-sensing instruments based on FBG strain sensors [25,58,59,60,61], which have been shown to accurately measure the micro-forces directly at the tool tip without significant signal degradation due to varying ambient temperature or the forces at the sclerotomy port. Following a similar approach, we developed a force-sensing microneedle with a thin (Ø 70 µm) bent (45°) tip, which is held straight in a protective shaft while inserting through the sclera and deployed out by means of a motorized unit upon reaching the retina surface [25]. Researchers from University of Leuven developed an alternative force-sensing needle also with a thin (Ø 80 µm) and bent (45°) tip independently from our previous work [24].

Combining a force-sensing cannulation tool with a robotic assistant is a potential solution to overcoming the challenges in RVC. Precisely identifying the moment of venous puncture and stably fixating the cannula throughout the drug delivery are both facilitated. We have previously attained an extended period of intravenous cannula stability in a dry phantom by combining a handheld micromanipulator, Micron, with a force-sensing microneedle [62], which has been, to the best of our knowledge, the first handheld assistive RVC system that can detect venous puncture and act automatically. This study builds on our prior work, discusses the development of the force-sensing microneedle tool in more detail, and presents a more extensive validation of the combined assistive system. In Section 2, we introduce the components of our system. Section 3 demonstrates our force and position based venous puncture detection methods and its integration with the micromanipulator control scheme. In Section 4 and Section 5, using an artificial phantom replicating the constraints of RVC, we evaluate the efficacy of the developed system for three operators with differing levels of experience on the robotic system and expertise in RVC. The paper concludes with a discussion of the results, current limitations and our future aims.

## 2. System Components

### 2.1. Motorized Force-Sensing Microneedle

#### 2.1.1. Actuation Mechanism

**Figure 1:**
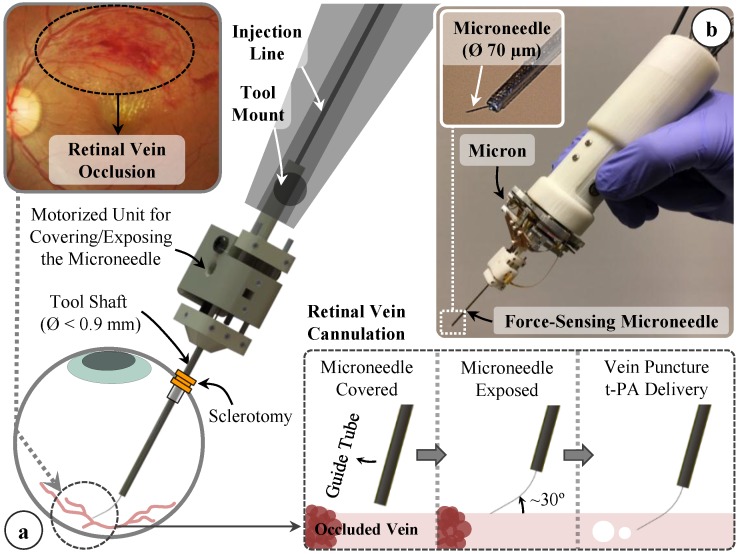
(**a**) Conceptual design of our motorized force-sensing microneedle for retinal vein cannulation; (**b**) The tool has a thin tip (Ø 70 µm) and a modular design that allows for easy integration with robotic devices, such as the Micron handheld micromanipulator.

**Figure 2:**
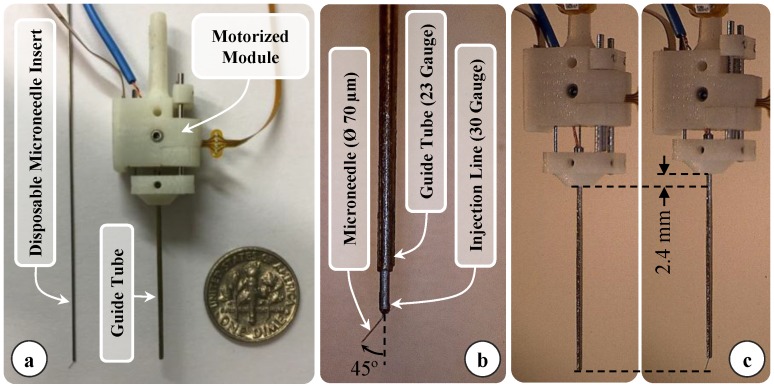
(**a**) Components of the design: force sensitive motorized unit, and the disposable prebent needle (**b**) Assembled prototype (**c**) Tool actuation: the microneedle is exposed by retracting the guide tube as much as 2.4 mm.

Injecting t-PA into thin branch retinal veins (Ø < 200 µm) requires the use of even thinner and sharp tipped cannulae. Targeting Ø 100–500 µm veins, we devised a motorized force-sensing microneedle with an outer diameter of 70 µm at the tip (Figure 1). This is a compact lightweight modular unit carrying all the necessary sensors (FBGs and an encoder) and a linear motor that can be independently actuated. This enables easy integration with various types of tool handles, either manual or robotic, without interfering with their operation. The tool has a sharp beveled (15°) and bent (45° relative to the tool shaft) tip. If a straight and blunt tipped microneedle were used, the vein walls would pushed closed. In addition, after piercing the vein wall, the instantaneous release of stress would cause the tissue to uncoil toward the needle, which would make overshooting the target vein almost inevitable. To avoid overshoot, it is important to approach the retina at an angle, and move the needle tip almost parallel to and with minimal vertical motion into the vein. This way, the tissue will deform less and along the vein axis, so that any relative motion due to tissue relaxation after venous puncture will not force the needle tip out of the vein lumen. Based upon these factors, the optimal approach angle while cannulating the vasculature on CAM was previously explored, which revealed a range of 25–35° between the needle tip and vein surface [23]. Due to constraints at the sclerotomy, it is not easy to approach the retinal surface at this angle with a straight tool. Using a flexible pre-bent tip can help, as long as the bending angle is carefully adjusted. Over bending the microneedle in order to achieve a very shallow approach angle may not only cause severe deformation of the needle while pushing it against the vein, but will also lead to excessive resistance to fluid flow inside the lumen at the bend, preventing injection. Considering these limitations, the approximate pars plana position for tool insertion, the dimensions of the typical adult eye, and targeting an ideal approach angle of about 30° [30], we bent the needle tip 45° relative to the tool shaft as shown in Figure 2b. The bent needle tip was bonded to a 30 gauge stainless steel tube using medical device adhesive (Loctite 4013 Prism, Henkel, Rocky Hill, CT, USA) to form the injection line of our tool. After mounting the tool onto a robotic device or a manual tool handle, the 30 gauge tube can be connected to a syringe controlled by a standard infusion pump (11 Pico Plus Elite, Harvard Apparatus, Holliston, MA, USA) via a sealed polyethylene tubing (PE-10, Warner Instruments) to deliver the injection fluid (t-PA as in [11] or ocriplasmin as in [12]).

**Figure 3:**
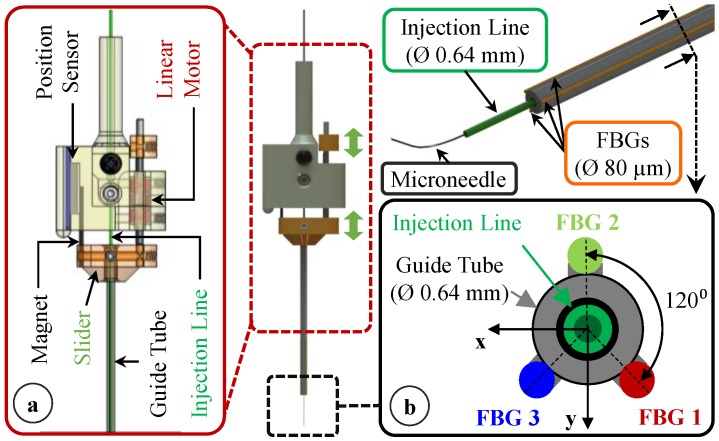
Motorized actuation of the tool for retracting the guide tube back and exposing the prebent microneedle. The inner tube of the tool shaft delivers the injection fluid, while 3 FBG sensors on the outer tube sense transverse forces on the microneedle. (**a**) The inner tube of the tool shaft delivers the injection fluid, while (**b**) 3 FBG sensors on the outer tube sense transverse forces on the microneedle.

Due to the bent shape of the thin needle, it is not easy to introduce the tool into the eye through a small incision (Ø < 1 mm) without damaging the tip. In order to keep the sharp and pre-bent microneedle protected and flexed straight while passing it through the sclera, we devised a motorized module with a retractable guide tube functioning as a protective sheath. As depicted in Figure 2a,b, the injection line (30 gauge tube with the bent microneedle attached at its tip) is passed through a 23 gauge guide tube and firmly anchored to the body of the module with the help of a set screw. This component is designed to be disposable since the needle tip will gradually lose its sharpness and impede venous puncture in the setting of consecutive cannulation attempts. By releasing the set screw, the old injection line with the dull tip can easily be replaced with a new one while preserving the rest of the module. The guide tube is fixed onto a slider mechanism driven by a linear micro motor (Squiggle-RV-1.8, New Scale Technologies Inc., Victor, NY, USA), so that when the motor is actuated, the guide tube moves along the tool shaft to cover or expose the needle tip. While inserting the tool into the eye and approaching the retina surface, the needle tip is flexed and held straight inside the guide tube. Upon reaching the target vessel site, retracting the guide tube deploys the bent needle tip (Figure 2c). Fully covering or exposing the microneedle requires a travel of 2.4 mm. The selected motor supplies enough force and travel range for this task in a very small (2.8 × 2.8 × 6 mm), and light weight (0.16 g) package. There is a bar magnet located on the side of the slider (Figure 3a). The position of the slider, and thus of the micro motor, is tracked via the magnetic position sensor (NSE-5310, ams AG, Unterpremstätten, Austria) located on the side of the base. This enables fine position control of the motor in closed loop (with a resolution of 0.5 µm and linear accuracy of 25 µm without loading), and ensures accurate motion of the guide tube despite the potentially variable friction at the insertion port through the sclera. The parts holding the guide tube, housing the motor, anchoring the injection line to the module and the lid shielding the slider mechanism were built using 3D printed acrylonitrile-butadiene-styrene (ABS) with a resolution of 0.254 mm. The assembled actuation unit occupies a space of 13.0 × 15.5 × 9.3 mm and weighs only about 2.9 g.

#### 2.1.2. Force Sensor Integration

Detection of the very fine tool-tissue interaction forces while cannulating retinal veins requires the use of a very sensitive force sensor with sub-mN resolution. In addition, the location of the force sensor is critical. Since the forces at the sclera insertion port can be much larger than the typical cannulation forces at the tool tip, a handle mounted force sensor measuring a combination of these two forces would not be appropriate. In order to capture only the tool tip forces, the sensor needs to be located proximal to the tool apex and inside of the eye. Bringing the sensor into the eye puts strict requirements and limits the available sensors for this use. FBG strain sensors (Technica S.A., Beijing, China) with their small dimension (Ø = 80 µm), high sensitivity, biocompatibility, sterilizability, and immunity from electrostatic and electromagnetic noise satisfy these criteria.

While inserting the needle into the vein, forces induced at the needle tip will be mostly along the axis of the needle, and hence will be dominantly perpendicular to the tool shaft due to the bent tip. Previously, our team has developed several ophthalmic tools capable of sensing the transverse forces at the tool tip with a 0.25 mN resolution during other retinal procedures [58,59,60,61]. Following a similar architecture, we bonded 3 FBG sensors evenly around the guide tube using medical device adhesive (Loctite 4013 Prism, Henkel, Rocky Hill, CT, USA) as shown in Figure 3b. This led to an overall tool shaft diameter less than 0.9 mm, which is sufficiently small for insertion through the sclera.

In order to transform the optical wavelength information from each embedded sensor to force values, we assume small elastic deformations and model the guide tube as an Euler-Bernoulli beam with transverse forces (within the plane formed by the x and y axes in Figure 3b) at the tip inducing a linearly proportional local elastic strain on each sensor, and thus producing a linearly proportional Bragg wavelength shift in each FBG. In addition, during vitreoretinal surgery, the intraocular temperature may show significant changes at different steps of the surgical procedure [63], which may induce a drift in sensor readings. In this case, the combined Bragg wavelength shift for each sensor is given by:

(1) $$\Delta \lambda_{i}=C_{i}^{F\_x}F_{x}+C_{i}^{F\_y}F_{y}+C^{\Delta T}\Delta T,\;i=1,2,3$$

where $C_{i}^{F\_x}$, $C_{i}^{F\_y}$ and $C^{\Delta T}$ are constants associated with the x, y, z forces and the temperature change, respectively. When the mean Bragg wavelength shift in all three lateral sensors is computed, due to axisymmetric distribution of lateral FBGs around the guide tube (120° apart from each other as depicted in Figure 3b), the terms related to the transverse forces in Equation (1) cancel each other as detailed in [58], resulting in the common mode ($\Delta \lambda^{\mathrm{mean}}$) which is a function of the temperature change only. The thermal drift in sensor readings can be eliminated by subtracting the common mode from each sensor’s wavelength shift as shown in Equation (2). The remaining differential mode of each sensor ($\Delta \lambda_{i}^{\mathrm{diff}}$) can then be used in Equation (3) to compute the forces at the tool tip. For transverse loads (F_x_ and F_y_), this temperature compensation method was previously shown for a retinal pick tool with similar force-sensing architecture to provide a robust response accurate within 2 mN when the tool was subject to thermal shocks [58]:

(2) $$\Delta \lambda_{i}^{\mathrm{diff}}=\Delta \lambda_{i}-\Delta \lambda^{\mathrm{mean}}=C_{i}^{F\_x}F_{x}+C_{i}^{F\_y}F_{y},\;i=1,2,3$$

(3) $${\left[\begin{array}{cc} F_{x} & F_{y} \end{array}\right]}^{T}=C\;{\left[\begin{array}{cc} \begin{array}{cc} \Delta \lambda_{1}^{\mathrm{diff}} & \Delta \lambda_{2}^{\mathrm{diff}} \end{array} & \Delta \lambda_{3}^{\mathrm{diff}} \end{array}\right]}^{T}$$

**Figure 4:**
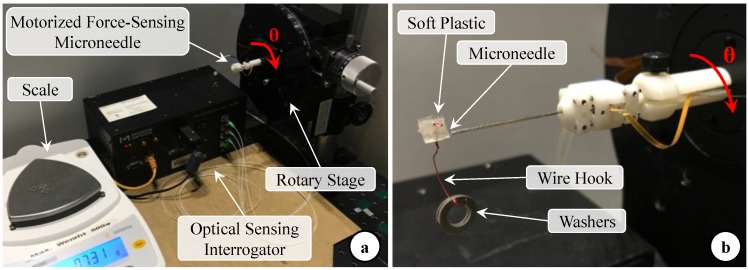
(**a**) Setup for calibration experiments: The force-sensing micro-needle was held horizontal and mounted on a rotary stage to modulate its axial orientation (θ); (**b**) The microneedle tip was inserted into a soft plastic piece carrying a wire hook. Forces were applied at the needle tip by hanging washers of known weight on the hook.

In Equation (3), C is a 2 × 3 coefficient matrix which represents the linear mapping from optical sensor readings to the force domain. In order to find this constant, we performed a calibration experiment using the setup shown in Figure 4a. We mounted our force-sensing tool horizontally on a rotary stage to modulate its axial orientation (θ). A soft plastic piece carrying a wire hook was attached to the microneedle tip (Figure 4b).

By hanging aluminum washers on the wire hook, varying forces (pure F_x_ at θ = 0° and pure F_y_ at θ = 90°) were induced at the tool tip. The washers were weighed using a precision scale (Sartorius GC2502, Göttingen, Germany) which provides a resolution of 0.01 mN and a repeatability of ±0.02 mN. The maximum test load was 20.6 mN, and each washer weighed about 1.03 mN. The Bragg wavelength of each FBG sensor was acquired using an optical sensing interrogator (sm130–700 from Micron Optics Inc., Atlanta, GA, USA). Measurements were taken for 20 different levels of loading at each orientation. For each test condition, 1000 samples were recorded at a rate of 200 Hz.

**Figure 5:**
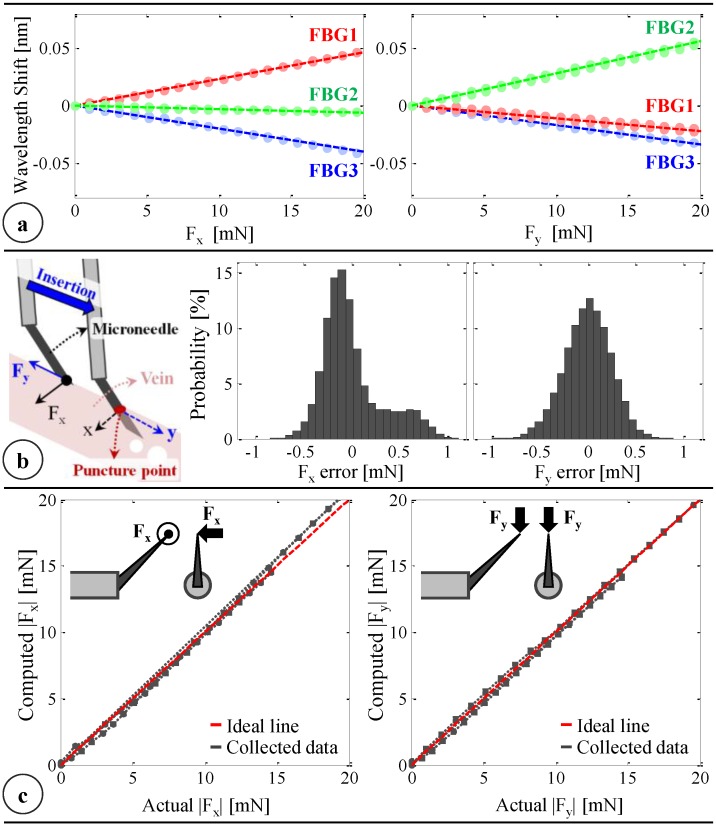
(**a**) Calibration results: linear response for all FBGs when the needle is loaded along x and y axes; (**b**) The force-sensing coordinates relative to the target vein and the histogram of residual errors in each direction; (**c**) Computed forces vs. the actual forces along the x and y axes [62].

The calibration results in Figure 5a show that all FBGs exhibit a linear reproducible behavior to forcing along both the x- and y-axis. Using this dataset, the linear system of equations given in Equation (3) is formed and solved by using the method of least squares to obtain the calibration matrix (C):

(4) $$C=\left[\begin{array}{ccc} -0.2128 & 0.2455 & -0.0327\\ -0.1418 & -0.0927 & 0.2346 \end{array}\right]\;\mathrm{mN}/\mathrm{pm}$$

The wavelength resolution of our optical sensing interrogator is 1 pm. Based upon the obtained calibration matrix, this corresponds to a resolution of about 0.25 mN for both F_x_ and F_y_. The optical sensing interrogator has a scan rate of 1 kHz, and the time constant of the sensor is about 5 ms [64], providing a fast enough response for accurate tracking of quick force variations in RVC. To validate sensor operation, the tool tip was loaded/unloaded repeatedly at three different orientations (0°, 45° and 90°). Twenty different load conditions (0–20.6 mN) were tested at each orientation, and each case was repeated six times (three load/unload cycles). For each test, 1000 samples were recorded at a rate of 200 Hz. Overall, the computed forces were observed to closely predict the actual forces with root mean square errors of 0.31 mN and 0.24 mN respectively for F_x_ and F_y_. The histograms of the residual errors (Figure 5b) show that the probability of errors beyond 0.5 mN is very low, especially for 0–10 mN range, where a closer fit to the ideal straight line (slope = 1) passing through the origin in Figure 5c is visible between the computed and actual forces. Above 10 mN, although F_y_ accuracy is preserved, F_x_ is observed to gradually deviate from the ideal line as the loading is increased. This also leads to higher order moments in the distribution of errors in F_x_ compared to the errors in F_y_ (Figure 5b), which can be attributed to the adverse effect of torque generated due to the distance between the tip of the bent needle and the tool shaft center. Such error can, in theory, be corrected by modeling the torsional effects on the force-sensitive elements. However, during RVC, since the needle insertion will take place mostly along the needle axis (y-axis of the tool), forces exceeding 10 mN along the x-axis are highly unexpected. Thus, the slightly bigger errors in predicting F_x_ at larger amplitudes are not a major concern.

#### 2.1.3. Effect of Actuation on Force Sensor Response

**Figure 6:**
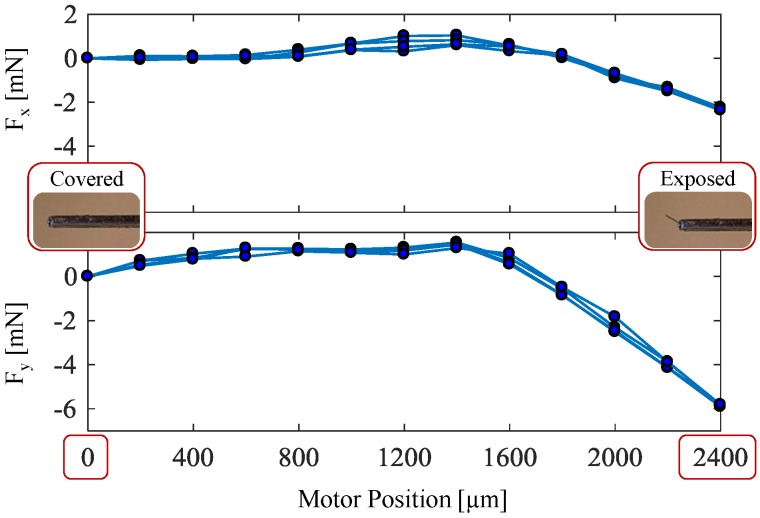
Effect of actuation on the sensed forces during three cycles of cover/expose cycles. Although the microneedle is not contacting any tissue, covering and exposing the microneedle shifts the force readings by about 2 mN in F_x_ and 6 mN in F_y_ with a consistent behavior. In practice, these undesired components due to actuation can be eliminated from the force readings by automatically rebiasing the FBG readouts once the motor position reaches the fully exposed position (2400 µm).

In this design concept, the force sensitive elements are integrated on a functional component that moves and goes under stress during the actuation of the tool. As shown in Figure 6, this causes a shift in FBG sensor readings, which produces an erroneous reading of about 2 mN in F_x_ and 6 mN in F_y_ when the guide tube is fully retracted back (motor position = 2.4 mm) although the needle tip is not interacting with the tissue yet. In addition, the computed calibration matrix is valid for the fully exposed configuration of the needle. The sensitivity may differ for middle positions and fully covered states, which are not relevant to the practical use of the needle. In our force computation algorithm, to get an accurate measurement of forces during cannulation, after the motor position reaches 2.4 mm and the needle is fully exposed, each FBG sensor is automatically rebiased to eliminate the actuation artifact and set a zero-force reference for the subsequent measurements. After touching the retinal surface, the interaction forces are computed relative to this baseline using the calibration matrix obtained in the previous section.

### 2.2. Integration with a Handheld Micromanipulation System: Micron

In order to suppress involuntary motion in retinal microsurgery, various robotic systems have been developed [27]. Among the handheld robotic devices is Micron [33], which aims to preserve the intuitive feel of standard handheld ophthalmic tools, and actively attenuate the physiological hand tremor of the surgeon. Micron has a custom optical tracking system, named Apparatus to Sense Accuracy of Position (ASAP). ASAP acquires the pose of the tool handle with 4 µm resolution and up to 2 kHz sampling rate, which is used to identify the voluntary and involuntary (tremulous) components of motion via a shelving filter. When activating its three piezoelectric actuators, Micron moves its tip to counteract the involuntary motion component within a workspace of approximately a 1 × 1 × 0.5 mm volume centered on the handle position. The control software for this operation mode is implemented in LabVIEW and can be altered to achieve other micromanipulation goals.

**Figure 7:**
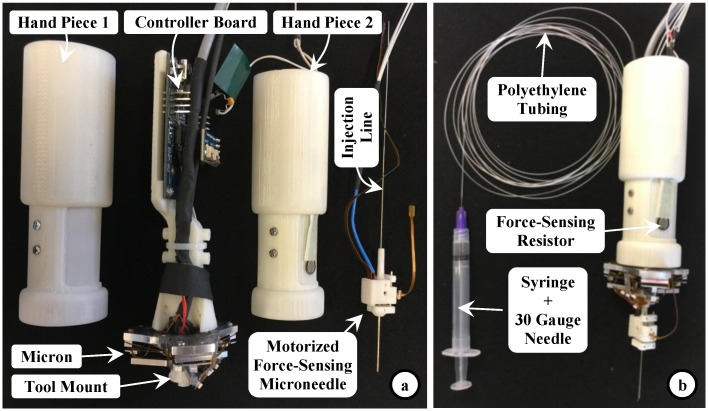
Sample integration of the motorized force-sensing microneedle with a handheld micromanipulator, Micron. (**a**) The controller board for the tool’s motor was located on Micron’s handle inside the two hand pieces; (**b**) A force-sensing resistor was mounted on one of the hand pieces, under the operator’s thumb, to control the microneedle’s actuation by squeezing or releasing the hand piece. The injection line passes through the center of the instrument and is connected to a syringe via polyethylene tubing to deliver fluid to the microneedle.

We attached our force-sensing motorized microneedle to Micron’s tool mount as shown in Figure 7. The fluid line (30 gauge tube protruding from the back of the tool) was passed through the hollow center of the Micron handle and connected to a syringe with a 30 gauge needle via a sealed polyethylene tubing (PE-10, Warner Instruments) to deliver the injection fluid. The infusion rate and amount can be controlling the syringe with a standard infusion pump (11 Pico Plus Elite, Harvard Apparatus). The controller board of the linear actuator was located inside the tool handle. A force-sensing resistor (FSR 400, Interlink Electronics, Westlake Village, CA, USA) was attached on the tool handle under the operator’s thumb and interfaced with the micro-motor controller such that the target position of the motor varies linearly with the exerted force on the resistor. Simply by releasing or squeezing the handpiece, the operator can cover or expose the microneedle, respectively.

## 3. System Operation

### 3.1. Control Scheme

Micron normally operates in a “Tremor Canceling Mode”, which attenuates physiological hand tremors by continually tracking the tool handle motion and filtering it to compute a goal position for the tool tip. This is a beneficial feature to accurately manipulate the microneedle inside the eye and aim the occluded vein during RVC. However, it is not sufficient to fully assist the procedure. After bringing the tip onto the target and while pushing it into the vein, identifying the moment of venous puncture is important to stop needle penetration in a timely manner—right after piercing through the superficial vessel wall and before piercing through the inferior vessel—to avoid double puncturing the vein. In addition, after this instant, any unintentional movement, not only the higher-frequency tremulous components but also the lower-frequency drift in tool position, needs to be counteracted to maintain the cannula inside the vein for a longer period with minimal trauma to the vasculature, until the infusion is completed. Based on the continual monitoring of acquired tool-tissue interaction forces via the devised force-sensing microneedle, and the tool tip position via Micron’s ASAP trackers, we developed two puncture detection algorithms, which were integrated with Micron’s software via a custom LabVIEW program leading to the new operational mode “Position Holding Mode”. In this control scheme, the wavelength information from each FBG sensor on the needle is collected and processed at 1 kHz and transmitted over TCP/IP to the LabVIEW environment. The tool-tissue interaction forces at the needle tip are computed based on the method and calibration matrix introduced in Section 2.1.2. Meanwhile, the ASAP trackers provide the needle tip position at a sampling rate of 1 kHz, which is used to compute tool tip velocity. Tool tip force and velocity are continually fed into our venous puncture detection algorithm, which will be detailed in the following section. Before the venous puncture occurs, Micron works in its regular “Tremor Canceling Mode”, filtering out the high-frequency components of the sensed tool motion, and therefore enabling a safe and steady approach to the target vein. When venous puncture is detected, an alarm sound is generated to inform the operator of the puncture event, and Micron switches from its “Tremor Canceling Mode” to the “Position Holding Mode” by locking its goal position to the tip position recorded at the instant of venous puncture. After the mode switch, even if the user unintentionally drifts the tool handle away, Micron deflects its tip to actively compensate the motion so that the tip is held fixed right at the puncture point. This mode helps fixate the needle inside the vein successfully as long as the drift of the user is within the limits of Micron’s actuators (within a sphere of ~0.5 mm radius). Deviating from the puncture point beyond these limits will saturate the actuators, and thus may not be compensated. Throughout the operation, the operator still maintains the gross positioning control while Micron makes fine adjustments to fixate the tool tip. In this way, Micron actuation can be overridden if needed, in case the needle needs to be quickly removed from the tissue.

### 3.2. Venous Puncture Detection

We investigated two different strategies for detecting venous puncture.

#### 3.2.1. Force-Based Method

In earlier needle puncture studies using rabbit ear veins [46] and CAM of fertilized chicken eggs [47], a characteristic force behavior was reported. After the needle tip touches the tissue surface, the tool-tissue interaction force gradually rises until a sharp drop signaling the entrance of the needle tip into the vein. Our first method aims to quickly identify this instant by continually checking the time derivative of forces measured by the sensitized microneedle. For this, a custom LabVIEW program was developed. During the operation, first, the Bragg wavelength of each FBG is obtained from the optical sensing interrogator, and using the calibration matrix, the transverse force at the tool tip is obtained. Then, the time derivative of tip force is computed and passed through a second-order low-pass filter. The optimal filter parameters (cutoff frequency and damping coefficient) are tuned based on measured force profiles in [47] so that the sharp oscillations due to measurement noise are filtered properly and the sharp drop in force is easily detectable with minimal delay (within 60 ms). When the filtered derivative of force turns negative ($d\left|{\vec{F}}_{\mathrm{needle}}\right|/\mathrm{dt}<0$), or falls below a certain threshold value (P) that distinguishes venipuncture from tool retraction ($d\left|{\vec{F}}_{\mathrm{needle}}\right|/\mathrm{dt}<P$), a puncture is reported by this algorithm, right after which the operator can be warned via an auditory signal or helped with a robotic assistance method to halt needle advancement and maintain its position fixed.

#### 3.2.2. Force-and-Position-Based Method

In cannulating retinal veins, manipulating the tool by hand either manually or with the aid of a robotic micromanipulator, it is difficult to guarantee a constant insertion speed. As shown in Table 1, while pushing the needle against the vein wall, moving the tool slightly slower or retracting it before the venipuncture can cause a decline in the force magnitude ($\left|{\vec{F}}_{\mathrm{needle}}\right|$), which can be misinterpreted still as a puncture event if the sensing system is checking only the sign of the exerted force’s time derivative ($d\left|{\vec{F}}_{\mathrm{needle}}\right|/\mathrm{dt}<0$). To distinguish a venous puncture from retraction events, a threshold value (P) needs to be specified such that only a sufficiently sharp drop in force ($d\left|{\vec{F}}_{\mathrm{needle}}\right|/\mathrm{dt}<P$) will be reported as a venous puncture. Identifying a proper threshold though requires careful tuning [24] and may differ depending on the local mechanistic properties of the inhomogeneous retinal tissue as well as the operator’s skill level. On the contrary, keeping track of both the force and position of the tool tip can give a more generalized and reliable prediction of the venous puncture without having to worry about the force derivative threshold.

Venous puncture is an event associated with a sharp drop in force while the needle is moving into the vein, which distinguishes it from a retraction event where the needle is moved away from the tissue. After the needle tip touches the vein wall, pushing it in will gradually deform the tissue and generate an increasing force at the needle tip. In contrast, retracting the needle backward will relax the tissue and therefore decrease the interaction force. Therefore, as long as the vein is not pierced, the law of action-reaction requires that the time derivative of the sensed tool tip force (${d\vec{F}}_{\mathrm{needle}}/\mathrm{dt}$) and the velocity of the tool tip (${d\vec{F}}_{\mathrm{needle}}/\mathrm{dt}$) always oppose directions; hence, the inner product of these two vectors is normally negative. Upon piercing the vein, however, this rule is reversed as the force variation vector (${d\vec{F}}_{\mathrm{needle}}/\mathrm{dt}$) instantly flips its orientation and points in the same direction as the velocity vector. Therefore, the venous puncture can be detected by continually checking the sign of the inner product of the force variation and velocity vectors, which will spike up to some positive value at the instant of venous puncture (${d\vec{F}}_{\mathrm{needle}}/\mathrm{dt}\cdot {d\vec{F}}_{\mathrm{needle}}/\mathrm{dt}>0$). Since our microneedle is bent 45° relative to the tool shaft, the insertion will induce mostly transverse (negligible axial) forces at the tool tip. Thus, although the force sensing properties of the microneedle are limited to only two dimensions ($F_{\mathrm{needle}}^{x}\;\mathrm{and}\;F_{\mathrm{needle}}^{y}$), puncture of the vein can still be captured effectively by taking ${\mathrm{dF}}_{\mathrm{needle}}^{z}/\mathrm{dt}=0$.

## 4. Experiments

**Figure 8:**
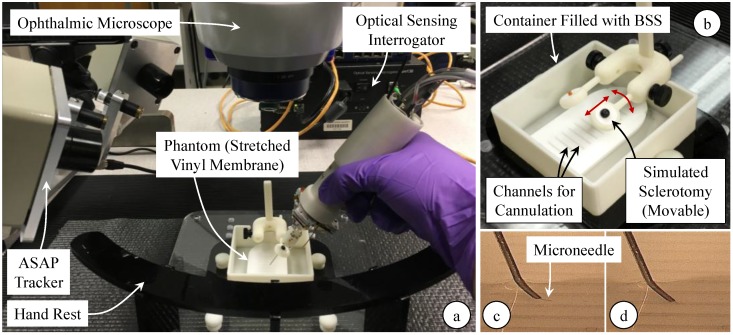
Experimental setup for cannulation experiments using an artificial phantom. (**a**) Stretched vinyl membranes simulating the vein walls were punctured using the force-sensing micro-needle under an ophthalmic microscope; (**b**) An acrylic insert with channels (1 mm wide) was used to tension the vinyl layer. The phantom was placed inside a container filled with balanced salt solution (BSS). To approach the channels on the phantom, the needle was inserted through the trocar on the movable (as shown with red arrows) sclerotomy port. A digital microscope was used to monitor the operational area from the side and assess the moment of puncture: (**c**) microneedle above the vinyl layer before puncture; (**d**) microneedle inside the channel after puncturing through the vinyl membrane.

The setup shown in Figure 8 was designed to replicate the main challenges of RVC, and as a very consistent platform to enable extensive testing. Due to its similarity in both scale and the composition of structure and morphology, CAM is an accepted in vivo model to study the retina and its vasculature [22]. However, using CAM, the number of trials for each test case remains limited, and it is hard to guarantee phantom consistency throughout the trials due to slight differences in the size of cannulated veins, and the anatomical variation between the eggs. To simulate the vein wall with consistent mechanical behavior, we created an artificial phantom with a thin membranous layer by stretching a vinyl layer (analogous to the polydimethylsiloxane sheet used in [29]) onto an acrylic insert with laser cut slots. The channels (1 mm wide) were cut much larger than the typical diameter of retinal veins to leave room for membrane deformation and unintentional drift of the operator’s hand without letting the needle tip contact the acrylic surface, and were made small enough to create support for the vinyl membrane’s surface tension.

Previous studies showed that the applied force while cannulating retinal veins can range anywhere from 5 mN to 35 mN depending on the angle of approach, the needle tip bevel, and the vein size [47]. The width of the acrylic insert in our phantom determines how stretched the vinyl membrane is, which affects the force required to pierce the membrane. The dimensions in the current setup produces a tension such that the piercing force varies within 5–15 mN. The phantom was placed inside a container, which was filled with balanced salt solution (BSS) to emulate the aqueous environment inside the eye after vitrectomy. A white silicone port (fabricated as outlined in [65]) was mounted above the phantom to simulate the sclera. To reach the phantom surface, the needle is first passed through the trocar on the silicone port. Analogous to the movable eyeball while manipulating the tools during surgery, the silicone port is not a hard constraint but can be rotated and translated as shown in Figure 8b. Experiments were performed under an ophthalmic microscope (OPMI Lumera, Zeiss, Oberkochen, Germany), providing only the top view of the phantoms. For analysis of collected data, a side view of the operation side (Figure 8c,d) was recorded using a digital microscope (USB2-Micro-200X, Plugable Technologies, Redmond, WA, USA).

Two types of experiments were conducted. In the first experiment, the phantom was punctured several times at different locations, aiming to verify phantom’s puncture properties and test the developed puncture detection methods. The experiment was performed by two subjects with differing skill and experience: (1) a non-surgeon with five years of experience on the robotic system but with no prior cannulation experience, (2) an ophthalmology fellow with moderate cannulation experience and 2 h of training on the robotic system. To prevent significant performance degradation in time due to fatigue, tests were completed in three periods, each period involving a total of eight trials, with a 10-min break between the periods. Similar to the procedural flow in RVC, the task in experiments was to puncture the vinyl membrane by moving the microneedle almost entirely laterally (in the direction the needle tip is pointing), and as slowly as possible without retracting the instrument before the puncture. After piercing the membrane—as observed from the side view of the operation site monitored by the digital microscope—the users were informed via auditory feedback, after which they tried to maintain the needle fixed for the following 45 s. Since each puncture causes the membrane to lose its tension locally, every new trial was done at a sufficiently distant location from the previous puncture point.

The second experiment aimed to quantify the effects of our system on operator performance during simulated cannulation trials using the same setup. The experiments were performed by three subjects with differing level of skill and experience: (1) a non-surgeon user with 5 years of experience on the robotic system but with no prior cannulation experience, (2) an ophthalmology fellow with moderate cannulation experience and 2 h of training on the robotic system, and (3) an expert vitreoretinal surgeon with 1 h of training on the robotic system. The task in each experiment was to approach the phantom submerged in the BSS solution, and puncture the vinyl membrane by moving the microneedle almost entirely laterally, in the direction the needle tip is pointing, and hold the needle fixed for 45 s after the event of puncture.

In the second experiment, two cases were explored by altering the operation mode of Micron: (1) only tremor canceling mode (TCM) versus (2) with the automatic position holding mode (PHM). In either case, the force-and-position-based puncture detection method from Section III.B was used, and the operator was informed upon the puncture via auditory feedback. The needle trajectory after this instant was recorded for analysis. To prevent significant performance degradation in time due to fatigue, tests were completed in three periods, each period involving a total of eight trials (four trials per case), with a 10-min break between the periods. Micron mode was altered between TCM and PHM in random order. Since each puncture on the phantom causes the membrane to lose its tension locally, every new trial was done at a sufficiently distant location from the previous puncture.

After completing the trials, the exact timing of each puncture was identified from the video recorded by the side-view digital microscope. In the first experiment, the recorded forces and tool tip position were used to test the feasibility of the force-and-position-based puncture detection method, and compare its performance to the force-based technique. In the second experiment, performance assessment was based upon the recorded deviation from the point of puncture. Analyses were done using one-way analysis of variance (ANOVA) followed by a *t*-test assuming unequal variance; statistical significance was defined as *p* < 0.05.

## 5. Results

### 5.1. Experiment 1: Evaluation of the Puncture Detection Methods

**Figure 9:**
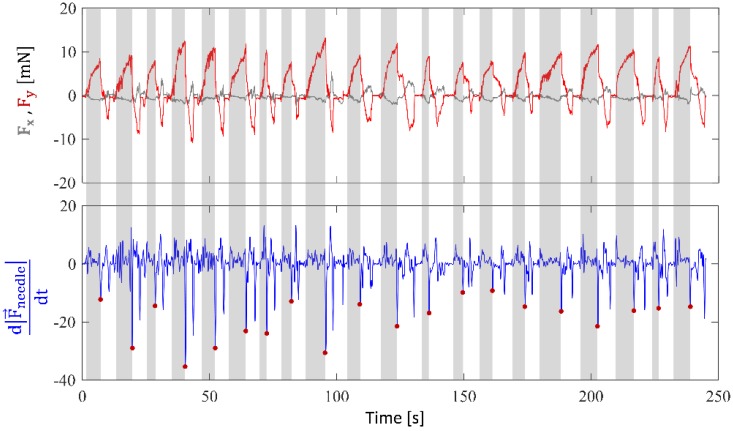
Measured forces during 20 consecutive punctures on the stretched vinyl membrane phantom. Grey zones correspond to the needle insertion phase while the white regions are for the retraction of the needle after puncture. During the insertion phase, most of the forces are along the needle axis (y-axis of the tool), with minor side load (F_x_ < 5 mN). The peak force before puncture ranges from 7.72 mN to 13.48 mN as the stretch of the phantom was adjusted targeting a range of 8–15 mN. The rate of force drop at the instant of piercing the membrane (marked with red dots in the lower plot) vary with a minimum of −35.21 mN/s and maximum of −8.73 mN/s for this set of trials.

Figure 9 shows the measured forces during 10 consecutive punctures at different locations on the phantom for the first user. Accordingly, the peak force (min: 7.72 mN, max: 13.48 mN) and rate of force drop at the instant of piercing the membrane (min: −35.21 mN/s, max: −8.73 mN/s) vary. The difference between punctures can be attributed to the slight discrepancies in the local surface tension of the membrane, variations in approach angle and insertion speed. In addition, there may be differences between the users. This makes it quite challenging to define a generalized threshold for detecting every puncture using the force-based method.

**Figure 10:**
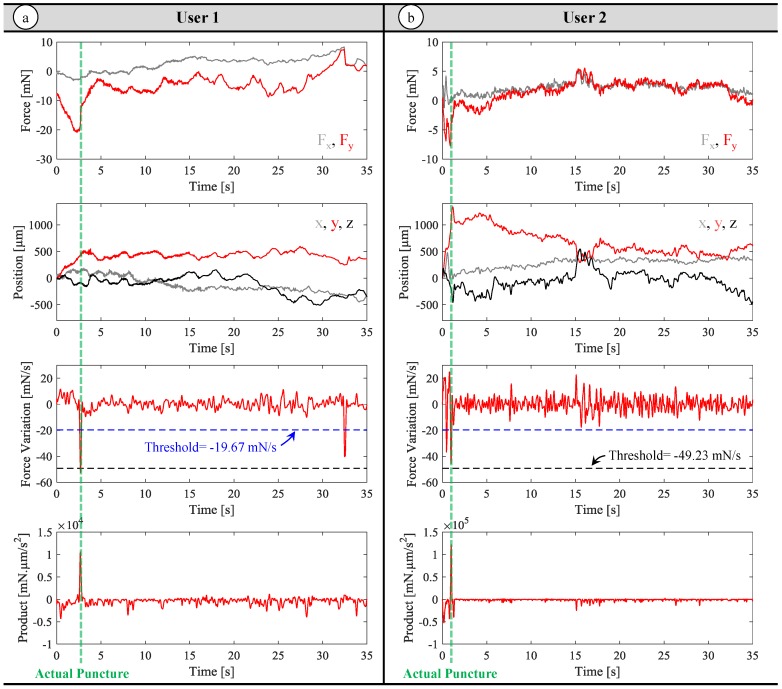
Measurements from a sample trial for each user: needle tip force (F_x_ in grey, F_y_ in red), needle tip position (x in grey, y in red, z in black), the variation of force ($d\left|{\vec{F}}_{\mathrm{needle}}\right|/\mathrm{dt}$) and the inner product of tool velocity and variation of force (${d\vec{F}}_{\mathrm{needle}}/\mathrm{dt}\cdot {d\vec{F}}_{\mathrm{needle}}/\mathrm{dt}$). The timing of the actual puncture in each trial is marked with a green dashed line. (**a**) Data for the first user: The elevated threshold (the dashed blue line at $d\left|{\vec{F}}_{\mathrm{needle}}\right|/\mathrm{dt}=-19.67\;\mathrm{mN}/s$) may intersect the time derivative of force multiple times, and may trigger faulty detections after the actual puncture. (**b**) Data for the second user: The low threshold (the dashed black line at $d\left|{\vec{F}}_{\mathrm{needle}}\right|/\mathrm{dt}=-49.63\;\mathrm{mN}/s$) fails to intersect the force variation curve while the elevated threshold intersects it twice, leading to a faulty detection before the actual puncture using the force-based method. In both trials, the inner product of the force variation and tool velocity clearly displays the instant of actual puncture with a prominent positive peak. The force-and-position-based method, relying on the inner product value, is able to distinguish the event of puncture.

The recorded force characteristics for each user and the performance of the developed puncture detection methods are summarized in Table 2. In addition, sample measurements from each user are presented in Figure 10. The exerted forces by the first user involve force drops ranging from −119.10 mN/s to −49.23 mN/s. On the other hand, the second user’s data reveals a wider range from −141.20 mN/s to −19.67 mN/s. Using the force-based method from the previous section, if the first subject’s data is taken as the basis for puncture detection ($d\left|{\vec{F}}_{\mathrm{needle}}\right|/\mathrm{dt}=-49.23\;\mathrm{mN}/s$, the black dashed line in Figure 10), then the first user’s all punctures can be accurately identified with only 2 trials involving a faulty detection after the actual puncture (false positives), which can be attributed to the involuntary movements and resulting force oscillations while trying to maintain the needle inside the membrane after the puncture. Despite the good results for the first user, this threshold is not appropriate for the second user’s performance as one third of the punctures, which have softer force drops, will be missed. The trial shown in Figure 10b exemplifies this issue, where the puncture leads to a force drop of $d\left|{\vec{F}}_{\mathrm{needle}}\right|/\mathrm{dt}=-45.51\;\mathrm{mN}/s$, and the threshold indicated by the dashed black line ($d\left|{\vec{F}}_{\mathrm{needle}}\right|/\mathrm{dt}=-49.23\;\mathrm{mN}/s$) does not intersect the force variation profile failing to detect the puncture. To remedy, if the threshold is set to a higher value ($d\left|{\vec{F}}_{\mathrm{needle}}\right|/\mathrm{dt}=-19.67\;\mathrm{mN}/s$), then all punctures for both users can be accurately sensed. Though among the 24 trials, in three of the trials in the second user’s performance, due to a momentary retraction of the needle before the actual puncture, the force profile follows a fluctuating trend rather than a steady rise, causing a faster force drop than the threshold value. This causes the force-based method with the elevated threshold to misinterpret these instances as puncture events. The sample trial shown in Figure 10b illustrates one such case, where the blue dashed threshold ($d\left|{\vec{F}}_{\mathrm{needle}}\right|/\mathrm{dt}=-19.67\;\mathrm{mN}/s$) intersects the force profile before the actual puncture due to the momentary retraction of the needle as evident from the trajectory plots. The retraction causes a force drop of $d\left|{\vec{F}}_{\mathrm{needle}}\right|/\mathrm{dt}=-36.76\;\mathrm{mN}/s$, which is under −19.67 mN/s and hence reported as a puncture by the force-based method. In addition, the elevated threshold triggers several false positives due to involuntary tool motion and rapidly fluctuating forces after the puncture for both users (in 83% of the trials for the first user, and 63% of the trials for the second user).

In contrast to the force-based method, monitoring the inner product of force variation and tool tip velocity gives a much clearer image, and helps with distinguishing between needle retraction and puncture events. As shown in the sample data for each user in Figure 10, the puncture leads to sharp positive peak in the product. Although the value of this peak can vary between users and trials, since the rest of variations in force and position lead to negative oscillations, the puncture, as the only prominent positive peak, remains very clear. As a result, using the force-and-position-based method, all punctures can be precisely detected for both users without any false positives before the actual puncture. This method performs satisfactorily as long as the filtered force and velocity information accurately describe the tool-tissue interactions. On the contrary, in the presence of rapid changes, the method can still lead to erroneous detection of punctures.

After the actual puncture, while trying to compensate the user’s drift and maintain the needle fixed in place, the micromanipulator can run out of its workspace (limited to 0.5 mm along each axis), which leaves no range to cancel user’s hand tremor. In this case, due to the tremulous tool motion and the complicated interactions while the tip is inside the membrane, the method will fail and report several faulty punctures. In terms of the faulty detections after the puncture, the force-based method with the lower threshold seems to have the best performance (Table 2), though it has the risk of failing to detect the actual puncture—only 67% of the actual punctures were detected for the second user. The force-and-position-based method can identify all the actual punctures for both users, and meanwhile provides much less frequent false detection after the puncture (46% and 50% of trials for users 1 and 2 respectively) in comparison to what the force-based method with the elevated threshold causes (83% and 63% of trials for users 1 and 2, respectively). These false positives can presumably be avoided by tuning the parameters of the low-pass filters on the velocity and force signals. However, this is not preferable as it would also induce a delay in predicting the actual puncture, and since for practical purposes, identifying the initial puncture precisely in a timely manner is of utmost importance. Without any false positives before the actual puncture and with a full detection rate of the actual puncture, the force-and-position based method has shown feasibility in predicting the instant of puncture quickly (within 90 ms) with less dependence on local mechanical properties of the phantom and the user’s performance.

### 5.2. Experiment 2: A Multi-User System Evaluation

**Figure 11:**
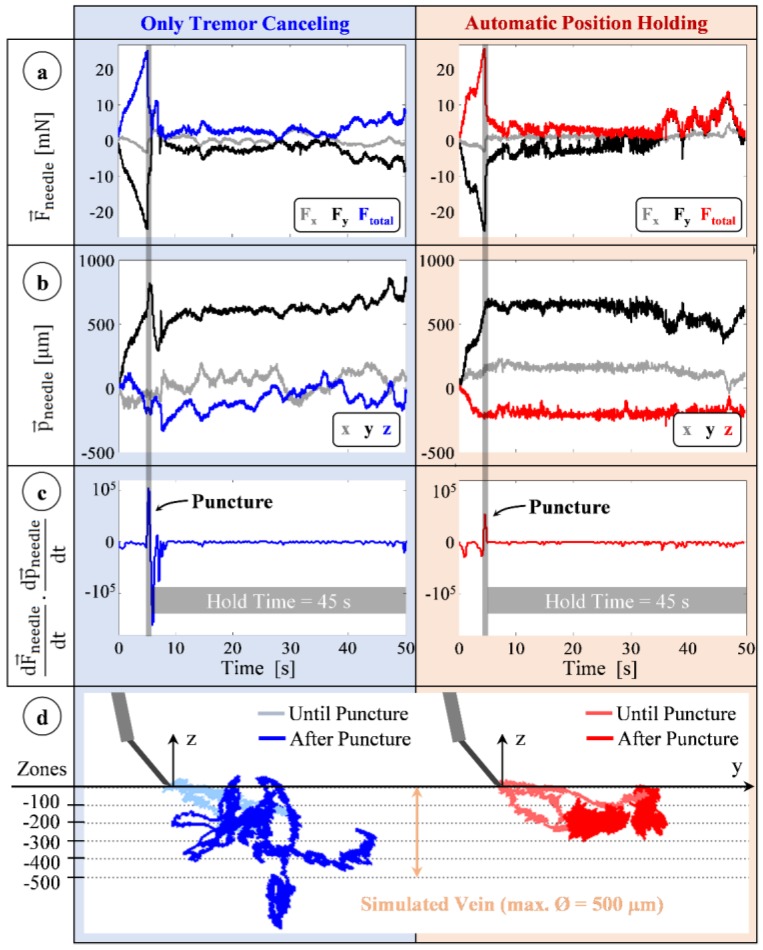
Typical measurements taken during trials of the first user using the tremor canceling (blue) and automatic position holding (red) features of the system. (**a**) Forces on the needle: mostly along the y-axis with a gradual rise during insertion and a sharp drop at the instant of puncture; (**b**) Position of the needle remains fixed with the position holding mode in contrast to the fluctuations with only tremor canceling; (**c**) The event of puncture: a positive peak in the inner product of needle velocity and force variation vectors; (**d**) Recorded needle tip trajectories for three representative trials of each mode. Position holding mode significantly reduced tool tip deviation from the puncture point [62].

Sample measurements from trials with TCM and with PHM for the first subject are shown in Figure 11. In these trials, sensed forces gradually rise after the microneedle touches the membrane surface (the simulated vein wall). Forces are mostly along the y-axis (all the measured x-axis forces remain under 5 mN) since the insertion is performed by moving the tool along the needle axis (y-axis) (Figure 5b). In both trials, the sharp positive peak in the inner product of needle velocity and force variation vectors (Figure 11c) shows the instant of puncture. During the 45 s period following puncture, there is a clear difference between TCM and PHM trials in terms of the tool tip travel, which is visible in Figure 11b. In TCM, all components of the position vector fluctuate, especially the z-component due to operator’s poor depth perception with the provided 2D microscope view.

Using PHM, the tool motion after the puncture is significantly lowered. Figure 11d presents three sample needle tip trajectories per operation mode for the first user, which shows a reduced deviation from the puncture point with PHM. The characteristics observed at the instant of puncture during the 24 trials for each user are summarized in Table 3. Accordingly, the puncture forces for all users lie mostly within the range that the phantom was developed for (5–15 mN). The mean value of the puncture force did not vary depending on the mode of operation (TCM or PHM), revealing no statistically significant difference (*p* = 0.83) between the mechanical behavior of membranes used in either test case. Furthermore, no statistically significant difference could be identified between the mean puncture forces of the three users (*p* = 0.12). The forces for the first user (F = 10.46 ± 1.70 mN), with extensive training on both the robotic system and the phantom, show the lowest deviation throughout the trials. Similar consistency is also observed in the surgeon’s (user 3) performance (F = 8.74 ± 1.70 mN). The rate of force drop associated with the event of puncture differs significantly among the users (−49.48 ± 15.75 mN/s for user 1, −67.47 ± 34.35 mN/s for user 2, −51.37 ± 16.06 mN/s for user 3, and *p* = 0.019). Therefore, to detect puncture, the force-based method would require separate tuning for each user. Using the force-and-position based method, the instant of puncture, which produced a clearly visible positive spike (8.75 × 10^3^–7.21 × 10^5^ mN·µm/s^2^) in the normally negative inner product of tool velocity and force variation, was detected precisely in all trials.

**Figure 12:**
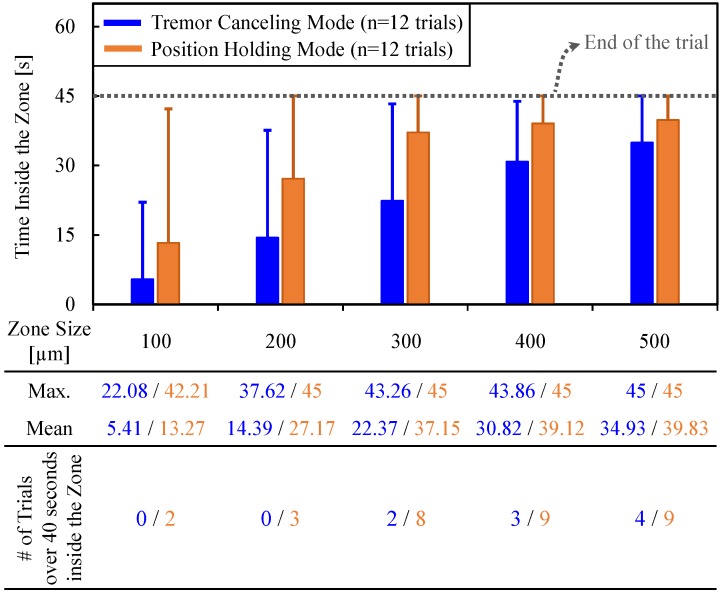
The total time that the needle tip was maintained inside various sizes of zones around the puncture point using the TCM (in blue) versus the PHM (in orange) features. The solid bars show the mean, and the error bars represent the maximum values. The active position holding helps in maintaining the needle tip inside the vein for a significantly longer time, especially for veins smaller than 300 µm [62].

To assess the ability to maintain cannulation in veins of different sizes, using the measured tool tip position throughout the entire hold period (45 s), we computed the time spent inside five zones of varying distance from the puncture point (Figure 11d). The size of each zone was chosen to represent a vein diameter, and was decided based upon the typical sizes of retinal vasculature (Ø 100–500 µm). In RVC, since both the vasculature and the microneedle are elastic, going beyond the borders of these zones does not necessarily mean that the needle will get out of the vein; instead, the needle will flex and the tissue will deform to some extent. The deviation from the puncture point, therefore, is a measure of the trauma that would have been induced on the vasculature in a clinical scenario. The results in Figure 12 show that, compared with the TCM trials, using the PHM enabled all the users to maintain the tip within each zone for a significantly longer period (*p* < 0.05). For instance, for the first user, who is the most experienced subject in using Micron and the phantom, the average time that the tip was maintained within a 100 µm distance from the puncture point was measured to be only 5.41 s with TCM; and in none of the 12 TCM trials could this duration exceed 40 s. In contrast, upon using PHM, the mean time was raised to 13.27 s, with 2 trials reaching a hold time over 40 s. Similar performance improvement with PHM was observed in the 200 and 300 µm zones. For the 400 and 500 µm zones, in terms of the average time maintained inside, no statistically significant difference between TCM and PHM could be identified for the first user (*p* = 0.07 for 400 µm, *p* = 0.26 for 500 µm). However, even for the 500 µm zone, where the performance of TCM and PHM is closest, the number of trials with successful “in-zone” hold time over 40 s was still 50% greater in PHM than TCM.

**Figure 13:**
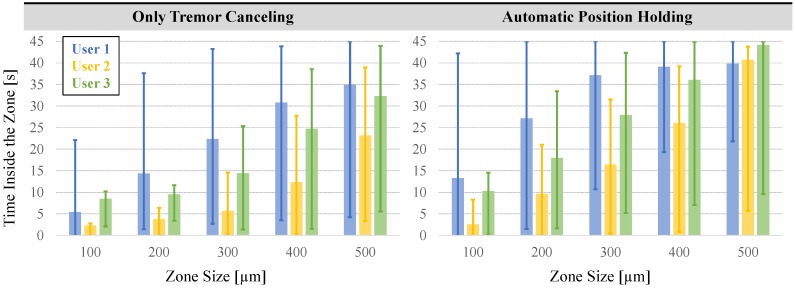
The performance of each user in maintaining the needle around the point of puncture (zone size = distance from the puncture point). User 1 is a non-surgeon with extensive experience on the robotic system. Users 2 and 3 are, respectively, an ophthalmology fellow and an expert vitreoretinal surgeon with some training on the robotic system. The solid bars show the mean, and the error bars represent the maximum and minimum values. The automatic position holding mode aids in maintaining the needle close to the puncture point for a longer period for all users.

The length of stay within each zone for all users with the TCM versus the PHM are compared in Figure 13. In contrast to the first user, the limited training that both user 2 (ophthalmology fellow) and user 3 (vitreoretinal surgeon) received on the robotic system is a factor affecting their performance. 

Despite that with TCM the third user could still maintain the needle inside the smallest zone (100 µm) for the longest time (8.47 s on the average) among others. As the zone size increases, the average time spent inside the zone grows for all users, following a different trend for each—with almost a constant rate for user 1, and with increasing rates for users 2 and 3. Nevertheless, the durations measured for all users, especially within the smaller zones, are far from being feasible to do prolonged infusions in RVC. When PHM is used, the average time spent in each zone is significantly improved for all subjects (*p* < 0.05). This shows that the PHM is helpful in keeping the needle close to the point of puncture for longer periods. For instance, user 3 could maintain the needle within 300 µm around the point of puncture for an average of only 14.43 s with the TCM, whereas with the aid of PHM this period was almost doubled up to an average of 27.92 s. For the largest zone (500 µm), all subjects exhibited almost identical performance using the PHM; the surgeon’s average time inside the zone (44.19 s) equaled almost the full test period. 

**Figure 14:**
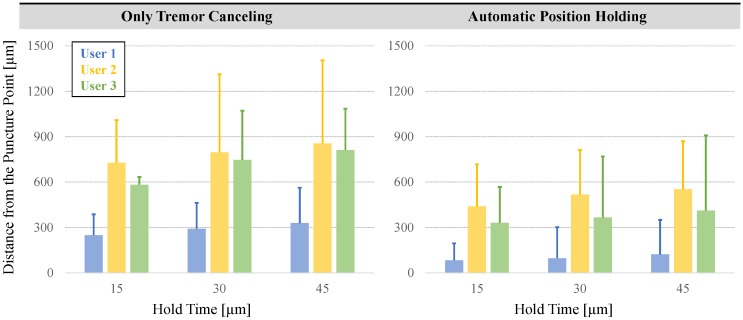
Deviation from the puncture point vs. the hold time for each user. The solid bars show the mean distance from the puncture point, and the error bars represent the maximum values. A longer hold time causes the needle to move away from the puncture point more. The automatic position holding mode significantly helps in keeping the needle tip proximal to the puncture point.

Our second performance metric is based on the range of needle tip motion after the puncture—which needs to be minimized to reduce trauma—as a function of the hold time. For this, we analyzed the tip deviation during the first 15, 30 and 45 s after the puncture for each user. The results in Figure 14 show that the maximum and mean deviation from the puncture point both increase as the needle is held for longer periods for all users, regardless of whether TCM or PHM was used. Nevertheless, for all hold times, both the mean and the maximum distance from the puncture point are significantly lower with the PHM for all users (*p* < 0.05). In case of a 15-s hold time for instance, the mean deviation from the puncture point for the first subject was reduced by more than 65% (from 276.18 µm to 90.34 µm). For the 30 and 45-s hold periods, the decay was similar. The mean deviation from the puncture point during a 45-second hold exceeded 350 µm with the TCM, whereas the PHM lowered it to below 200 µm for user 1. Similarly, for the second and third users, the mean drift after the puncture was reduced from 854.98 µm to 553.06 µm and from 812.06 µm to 412.32 µm, respectively. These results show that the automatic position holding is an essential feature that can potentially enable the cannulation of smaller veins, and provide an extended period of intravenous cannula stability, and thereby allow sustained periods of drug delivery at tolerable pressures.

## 6. Conclusions

Retinal vein cannulation is a demanding procedure, and its feasibility is currently limited by the challenges in identifying the moment of venous puncture, achieving cannulation and maintaining cannulation during drug delivery. In this study, we addressed these problems using a force-sensing microneedle combined with a handheld robotic micromanipulator, leading to two main contributions. First, to identify the instant of venous puncture, two distinct methods were developed and investigated. The first method based on the time derivative of only the tool tip forces required careful tuning of a threshold value that distinguishes the puncture from other force drops for each user. The second method which made use of both the tool tip force and position information together, and monitored the inner product of force variation and tool velocity vectors, revealed a more reliable prediction of the puncture without requiring the adjustment of any user-dependent parameters. The second contribution of this study is an automatic position holding feature which was developed to switch from the default tremor canceling operation mode of the micromanipulator to a full motion compensation. After identifying the venous puncture, the robotic system with this new feature helped in keeping the cannula fixed at the detected puncture position. Using an artificial phantom, we conducted a multi-user study with subjects from three different levels of skill and experience evaluating the performance of the Micron system with and without this new feature. Results showed that the developed puncture detection algorithm combined with active positive holding can maintain the needle tip inside the vein for a much longer time especially for smaller veins, and that it significantly attenuates the tool motion in the vein following venipuncture, thereby reduces the trauma on the tissue during cannulation.

The evaluation studies presented in this paper are based upon a fixed phantom position, and are limited to the very consistent mechanical behavior of an artificial phantom. Our future work aims to extend this evaluation by involving more users, collecting subjective user feedback in addition to quantitative performance metrics, replacing the handheld micromanipulator with a newer version with larger workspace, and integrating the effect of variability in tissue properties using in vivo models, which may reveal more distinguishing results in the performance of novice and expert operators. In a real clinical scenario, after venous puncture, the cannula needs to be maintained fixed relative to the tissue. Therefore, it is essential to account for potential movement of the tissue. Force information from the sensitized cannula may be used to detect relative movements and stabilize the cannula relative to the vasculature. Alternatively, optical coherence tomography guidance can be used after integrating an additional imaging fiber on the sensitized cannula to track and fixate tool-tissue distance after the venous puncture, which are some of the potential future directions.

## Figures and Tables

**Table 1:** Variation of tool tip force and position before and at the instant of puncture.

Before Puncture		At the Instant of Puncture
$\frac{d\left|{\vec{F}}_{\mathrm{needle}}\right|}{\mathrm{dt}}>0$	$\frac{d\left|{\vec{F}}_{\mathrm{needle}}\right|}{\mathrm{dt}}<0$	$\frac{d\left|{\vec{F}}_{\mathrm{needle}}\right|}{\mathrm{dt}}<P$
$\frac{{d\vec{F}}_{\mathrm{needle}}}{\mathrm{dt}}\cdot \frac{{d\vec{F}}_{\mathrm{needle}}}{\mathrm{dt}}<0$	$\frac{{d\vec{F}}_{\mathrm{needle}}}{\mathrm{dt}}\cdot \frac{{d\vec{F}}_{\mathrm{needle}}}{\mathrm{dt}}<0$	$\frac{{d\vec{F}}_{\mathrm{needle}}}{\mathrm{dt}}\cdot \frac{{d\vec{F}}_{\mathrm{needle}}}{\mathrm{dt}}>0$
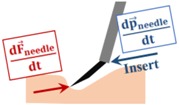	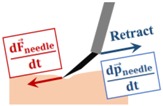	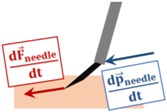

**Table 2:** Puncture detection rates with the force-based versus the force-and-position-based methods for two users with differing skill and experience (n = 24 trials per user).

Puncture Criterion	Force-Based Method						Force-and-Position-Based Method		
	$\frac{d\left|{\vec{F}}_{\mathrm{needle}}\right|}{\mathrm{dt}}<-49.23\;\mathrm{mN}/s$			$\frac{d\left|{\vec{F}}_{\mathrm{needle}}\right|}{\mathrm{dt}}<-19.67\;\mathrm{mN}/s$			$\frac{d{\vec{F}}_{\mathrm{needle}}}{\mathrm{dt}}\cdot \frac{d{\vec{p}}_{\mathrm{needle}}}{\mathrm{dt}}>0$		
	Detected the Actual Puncture	False Detection Before Puncture	False Detection After Puncture	Detected the Actual Puncture	False Detection Before Puncture	False Detection After Puncture	Detected the Actual Puncture	False Detection Before Puncture	False Detection After Puncture
**User 1**	100%	0%	8%	100%	0%	83%	100%	0%	46%
**User 2**	67%	0%	17%	88%	13%	63%	100%	0%	50%

**Table 3:** Force characteristics associated with the instant of puncture for three different users (n = 24 trials/user).

	$|{\vec{F}}_{\mathrm{needle}}|$				$\frac{d\left|{\vec{F}}_{\mathrm{needle}}\right|}{\mathrm{dt}}\;\left[\mathrm{mN}/s\right]$				$\frac{d{\vec{F}}_{\mathrm{needle}}}{\mathrm{dt}}\cdot \frac{d{\vec{p}}_{\mathrm{needle}}}{\mathrm{dt}}\;\left[\mathrm{mN}\cdot µm/s^{2}\right]$			
	MEAN	STD.	MIN.	MAX.	MEAN	STD.	MIN.	MAX.	MEAN	STD.	MIN.	MAX.
**User 1**	10.46	1.70	7.68	12.09	−49.48	15.75	−78.31	−26.14	7.08 × 10^4^	4.18 × 10^4^	1.53 × 10^4^	1.34 × 10^5^
**User 2**	11.66	6.90	4.55	22.45	−67.47	34.35	−141.20	−19.67	2.07 × 10^5^	1.61× 10^5^	8.75 × 10^3^	7.21 × 10^5^
**User 3**	8.74	1.70	6.51	13.02	−51.37	16.06	−89.21	−29.83	8.17 × 10^4^	4.53 × 10^4^	2.62 × 10^4^	1.69 × 10^5^
